# Effects of Dietary Supplementation With *Clostridium butyricum* on Growth Performance, Apparent Digestibility, Blood Metabolites, Ruminal Fermentation and Bacterial Communities of Fattening Goats

**DOI:** 10.3389/fnut.2022.888191

**Published:** 2022-05-24

**Authors:** Chengrui Zhang, Qingyuan Yu, Jihong Wang, Yidong Yu, Yonggen Zhang, Yukun Sun

**Affiliations:** ^1^College of Animal Science and Technology, Northeast Agricultural University, Harbin, China; ^2^Ordos Academy of Agriculture and Animal Husbandry, Ordos, China

**Keywords:** *Clostridium butyricum*, ruminants, fattening goats, growth performance, microbiota

## Abstract

*Clostridium butyricum* (*C. butyricum*) is currently widely used to improve the body health and productive performance of monogastric animals. However, there have been few reports on the effects and specific mechanism of action of *Clostridium butyricum* in ruminants. This study aimed to investigate the effects of *Clostridium butyricum* supplementation on the growth performance and digestive microbiota of fattening goats. Twenty-four healthy male Albas goats (body weight = 22 ± 2.03 kg) were randomly divided into 3 treatment groups with eight goats in each group. The treatments were as follows: control group (CON) (basal diet, concentrate to forage ratio = 65:35); low-dose *Clostridium butyricum* (LCB) (basal diet plus 2.0 × 10^8^ CFU/kg *Clostridium butyricum*); and high-dose *Clostridium butyricum* (HCB) (basal diet plus 1.0 × 10^9^ CFU/kg *Clostridium butyricum*). The experiment lasted for 8 weeks after a 2-week adaptation period. Therefore, growth performance and rumen and rectum microbiota were evaluated in goats supplemented with *Clostridium butyricum* and its metabolites. The results showed that dietary supplementation with *Clostridium butyricum* significantly increased the pH (*P* < 0.05), but had no significant effect on growth performance (*P* > 0.05). Compared with the control group, dietary *Clostridium butyricum* supplementation significantly increased the relative abundance of *Prevotella_1, Christensenellaceae AE_R-7_Group* and *Prevotellaceae AE_UCG-003* (*P* < 0.05), and significantly decreased *Succiniclasticum* and *Muribaculaceae_unclassified* (*P* < 0.05). The relative abundance of *Clostridium* in the rumen was <1.0%. Moreover, 16S rDNA analysis showed that the fecal *Clostridium* or *Clostridium butyricum* count was significantly decreased (*P* < 0.05), and the relative abundance of *Alistipes* and *Akkermansia* was increased (*P* < 0.10) in the low-dose group compared with the control group. Supplementing *Clostridium butyricum* in a high-concentrate diet did not significantly affect the performance of goats, while regulation of the gastrointestinal microbiota and related metabolites was associated with rumen fermentation.

## Introduction

The improvement of people's living standards and high consumption levels have contributed to the increasing worldwide demand for meat, eggs and milk (1). Short-term high-concentrate feeding can increase the growth rate of fattening sheep in ruminant production. There has been increasing interest in identifying novel approaches, such as improving feed digestibility, eliminating anti-nutritional factors and improving the metabolic level of animals, to improve feed efficiency and promote growth (2, 3). However, the specific mechanism of action of probiotics as a feed additive to promote the growth performance of ruminants is still unclear.

*Clostridium butyricum* (*C. butyricum*) is a gram-positive endophytic bacterium with anaerobic probiotic properties and high tolerance to the gastrointestinal environment due to its independent digestive enzyme system. It can produce a variety of substances, such as enzymes (4), vitamins (5), small peptides (6) and other metabolites, of which butyric acid, as one of its products, can provide the host with energy (7). Butyric acid can promote the development of gastrointestinal epithelial tissue (8) and consolidate its protective barrier function (9), facilitate the digestion and absorption of feed, and promote growth. Several studies have reported that volatile fatty acids (VFAs) play important roles in metabolism and intestinal microflora. In addition, other metabolites of *C. butyricum*, such as teichoic acid, promote *C. butyricum* colonization in the intestinal tract due to their highly adhesive properties (10). *C. butyricum* secretes digestive enzymes to improve the feed conversion rate in the gastrointestinal tract (4), and micropeptide substances (bacteriocin) competitively adhere to the surface of the intestinal epithelium and inhibit the invasion of pathogenic bacteria (6). These secretions regulate the structure and composition of bacteria *in vivo*, improve the homeostasis of the internal environment and promote the growth and development of livestock.

As the main metabolites of *C. butyricum*, butyric acid, bacteriocin and enzymes have been reported to improve antioxidant capacity, relieve inflammation, regulate intestinal immune function and gastrointestinal barrier function in mouse models and human subjects. Supplementation with *C. butyricum* in piglet and poultry diets has been associated with increased growth performance in animal production and can maintain homeostasis of the intestinal environment (4, 11, 12). Meanwhile, *C. butyricum* can improve antioxidant and immune functions in calves and monogastric animals (4, 13, 14). Numerous studies have also shown that *C. butyricum* can regulate the relative abundance of intestinal flora, consolidate intestinal barrier function and accelerate metabolism, to improve bodily health and immunity (12, 15, 16). In addition, its metabolite butyrate and other substances are also widely used in animal production to improve the performance of livestock and poultry, including improving the rumen fermentation of ruminants, and regulating rumen flora (17, 18). High-concentrate diets in production can provide higher energy in the short term for increasing ruminant growth rates. During the fattening period, it is necessary for goats to consume high-concentrate diets. However, the high-concentrate diets can lead to a rapid decrease in rumen pH, which can cause ruminal acidosis or subacute ruminal acidosis (19), thereby affecting rumen homeostasis and possibly goat growth performance. *C. butyricum* as a probiotic may have the potential to improve rumen homeostasis by modulating the rumen pH and microbiota, but what is the effect on the rumen microbiota is unknown. And there have been few reports on the effect and specific mechanism of action of *C. butyricum* in ruminants.

Therefore, we hypothesized that *C. butyricum*, as a feed additive, could influence the rumen environment through the activities of its active metabolites. The present study evaluated, the effects of dietary supplementation with *C. butyricum* on the growth performance and digestive microflora of fattening goats.

## Materials and Methods

All experimental designs and protocols were approved by the Institutional Animal Care and Use Committee of Northeast Agricultural University (Harbin, China) (Protocol number: NEAU- [2011]-9) and followed the recommendations of the academy's guidelines for animal research.

### Animals, Experimental Design and Diets

Twenty-four male Albas goats [body weight (BW)= 22 ± 2.03 kg] were divided into three treatment groups with eight goats in each group in a completely randomized trial design. The treatment groups were fed a control diet (CON), low-dose *C. butyricum* (LCB) or high-dose *C. butyricum* (HCB) with a diet of 0, 2 × 10^8^, or 1 × 10^9^ CFU/kg *C. butyricum* per goat per day, respectively. The basal diet ration was concentrate and forage, provided separately. The concentrate to forage ratio of the diet was 65:35, which was designed according to feeding standards for meat-producing sheep and goats (NY/T 816-2004, Ministry of Agriculture, China). The concentrate was commercial pellets (provided by Jiuzhou Dadi Feed Co., Ltd., Inner Mongolia, China), and the roughage was mixed forage consisting of oats and alfalfa used in the pasture (oats: alfalfa = 3:7). *C. butyricum LXKJ-1* was provided by Lvxue Biotechnology Co., Ltd. (Hubei, China; effective colony count ≥1 × 10^9^ CFU/g). The ingredients and nutritional composition of the diet are shown in Table 1.

**Table 1:** Composition and nutrient levels of diets (DM basis, %).

**Ingredients**	**Contents, % (DM basis)**	
	**Concentrate**	**Mixed forage^a^**
Corn	40	
Corn germ meal	20	
Shotcrete corn husk	13	
DDGS^b^	10	
Extruded soybean	8	
Molasses	3	
Limestone	4	
NaCl	1	
Compound premix^c^	1	
Total	100	
Nutrient levels^d^
Dry matter	90.38	92.04
Crude protein	18.93	11.67
Ether extract	4.72	2.15
Crude ash	6.02	7.69
Neutral detergent Fiber (NDF)	17.58	55.75
Acid detergent fiber (ADF)	6.01	35.39
Metabolizable energy (MJ/kg)	14.09	14.05

a *Mixed forage, oats to alfalfa is 5:5*.

b *DDGS, distillers dried grains with solubles*.

c *Each kilogram of composite premix includes: Ca 1.54 g, P 0.51 g, Fe 25 mg, Zn 35 mg, Cu 8 mg, Co 0.1 mg, I 0.9 mg, Se 0.25 mg, Mn 19.5 mg, VE 1000 IU, VA 3000 IU, VD 1000 IU*.

d *ME was a calculated value, while the others were measured values*.

The experiment was carried out on a family ranch in Otoki (Ordos, China). Each goat was individually kept in a pen with free access to water. *C. butyricum* was weighed daily according to the feed intake of each goat and then fed directly into their mouth per day, ensuring full intake. Daily supplementation with *C. butyricum* was adjusted according to the feed intake of the goats. The basal diet was fed twice daily at 06:00 and 18:00. Before formal feeding, all animals were fed albendazole (10.8 mg/kg BW) to expel parasites. The concentrate to forage ratio was adjusted by feeding a basal diet, and the adaptation period was 14 days. Treatment was initiated on Day 15 and lasted for 56 days.

### Feed Nutrient Composition

The feed samples were dried in an oven at 55°C for 48 h and then moistened for 24 h. They were crushed to pass through a 1-mm screen, sealed in a 150 × 220 mm sealed bag and stored at 4°C for the determination of nutritional components.

Wet chemical analysis was used to determine dry matter (DM, 934.01) and ash (Ash, 938.08) contents in all feed raw materials and fecal samples according to the analytical procedure of the Association of the Official Analytical Chemists (20). Crude protein (CP, 954.01), crude fat (ether extract, EE, 920.39), neutral detergent fiber (NDF) and acid detergent fiber (ADF) were analyzed according to the method of Van Soest et al. (21). NDF was assayed by thermally stable amylase. NDF and ADF results were expressed based on DM, including residual ash.

### Growth Performance

On Day 14 of the prefeeding period, all goats were weighed before the morning feeding to obtain the initial weight. Similarly, all goats were weighed before the morning feeding on Day 70 to obtain the final weight and calculate the average daily gain (ADG). Feed intake and residue were recorded daily to calculate total feed intake.

### Apparent Digestibility of Nutrients

Fecal and urine samples were collected on Days 51, 52, and 53 of the experimental periods. Samples from each goat were mixed for three consecutive days, and fecal and urine samples were divided into 2 parts. Approximately 50 ml of 6 mol/L sulfuric acid was added to one of the fecal samples, and approximately 10 ml of 0.036 mol/L sulfuric acid was added to the urine sample for the determination of nitrogen content. All fecal samples were dried in an oven at 55°C for 48 h and then moistened for 24 h. They were crushed through a 1 mm screen, sealed in a 150 × 220 mm sealed bag and stored at 4°C for the determination of nutrient digestibility. One additional fecal sample was immediately frozen in liquid nitrogen and stored at −80°C after being transferred to the laboratory for the determination of fecal microbes.

The digestibility of NDF, CP and EE in feed and fecal samples of all goats was estimated based on the acid insoluble ash (AIA) that and determined according to the 3 mol/L HCl insoluble ash described by Thonney (22) and method (GB/T23742-2009). Samples were dried to a constant weight at 55°C to determine the DM content. NDF, CP and EE were determined by an automatic fiber analyzer, Kay nitrogen analyzer and Soxhlet extraction system, respectively. The AIA method was used to calculate the apparent digestibility with the following formula:


$$\begin{array}{l} Apparent\;digestibility\;(\%)\;=100-[(M2n\times M1m)\\ \;/(M1n\times M2m)] \end{array}$$

where M_1m_ is the AIA content in the diet (%), M_2m_ is the AIA content in the feces (%), M_1n_ is a certain nutrient content in the diet (%), and M_2n_ is the nutrient content in the feces (%).

The calculation formula of feed conversion ratio (FCR) is as follows:


$$\begin{array}{l} FCR\;\left(kg/kg\right)\;=\;\left(Total\;feed\;intake\;/\;weight\;gain\right) \end{array}$$

The creatinine (CREA) content in urine was determined using a commercial kit (Nanjing Jiancheng Institute of Biological Engineering), which was operated according to the kit instructions. According to Valadares (23) and Leonardi et al. (24), the creatinine output was set at 29 mg/kg to calculate fecal nitrogen, urine nitrogen, nitrogen intake (NI), nitrogen absorption (NAB), nitrogen retention (NR) and net protein utilization (NPU). The calculation formula is as follows:


$$\begin{array}{l} Fecal\;N\;(g/d)\;=\;(Intake\;CP\;(g/d)\;Intake\;CP\;(g/d)\\ \;\times \;CP\;apparent\;digestibility\;\%)/6.25 \end{array}$$

$$\begin{array}{l} Urine\;N\;\left(g/d\right)\;=\;urine\;volume\;\left(L/d\right)\;\times \;urine\;CP\;\left(g/L\right)\;/\;6.25\\ NI\;\left(g/d\right)\;=\;Feed\;intake\;\left(kg/d\right)\;\times \;CP\;\%\;in\;feed\;\times 1000\;/\;6.25 \end{array}$$

### Blood Collection and Analyses

Approximately 15 ml of blood was collected from the jugular vein of each goat into heparinized tubes. The anticoagulant plasma was generated by centrifugation at 3,500 x *g* for 15 min and stored at −20°C for plasma biochemical analysis (33). Plasma total protein (TP), albumin (ALB), globulin (GLB), glucose (GLU), urea (BUN), creatinine (CREA), aspartate aminotransferase (AST), alanine aminotransferase (ALT), triglyceride (TG), total cholesterol (T-CHOL), low-density lipoprotein (LDL) and high-density lipoprotein (HDL) concentrations were analyzed using an automatic biochemical analyzer (HT82-BTS-330, Xihuavi Technology Co., Ltd., Beijing, China).

### Ruminal Fermentation Parameters

Two hours after feeding, four goats from each group were randomly selected and rumen fluid samples were collected by inserting gastric tubes down the esophagus. We discarded the first sample of fluid to reduce contamination with saliva (25). The collected rumen fluid samples were filtered through 4 layers of gauze for pH determination (pH meter: HI9125; Hanna Instruments, Padova, Italy). Then, 5 ml rumen fluid samples were divided into two 10 ml centrifuge tubes, and 1 ml metaphosphate solution (25%, W/V) was added into each tube. After mixing, the samples were stored at −20°C for the later determination of volatile fatty acids (VFAs) and ammonia nitrogen (NH_3_-N). Finally, the samples were divided into two 5 ml cryo-storage tubes, frozen with liquid nitrogen, transferred to the laboratory and stored at −80°C for later rumen microbial analysis.

VFA was determined by gas chromatography (Shimadzu GC-2010, Kyoto, Japan). Different concentrations of VFA standard solution were prepared and the content of VFA in the fermentation broth was determined using a standard curve method. The sample was centrifuged at 10,000 x *g* for 15 min, and the supernatant was filtered using a water filtration membrane, sealed and stored at 4°C for gas chromatographic determination. The injector and detector temperatures were 220°C. The initial temperature was maintained at 120°C for 3 min, then increased by 10°C/min to 180°C. The carrier gas was high-purity nitrogen. The injector pressure was maintained at 90 kPa, the hydrogen flow was 40 ml/min, the air flow was 400 ml/mi and the tail blowing flow was 45 ml/min.

NH_3_-N was determined by the indophenol colorimetric method (26). The collected rumen fluid samples were centrifuged at 12,000 *g* for 20 min, and the supernatant was taken for analysis. Approximately 40 μl of sample or standard solution was added to the test tube. Distilled water was used as a control. Approximately 2.5 ml of phenol solution was added to the sample and mixed thoroughly. Absorbance was measured at 550 nm using a spectrophotometer after heating in a water bath at 37°C for 30 min.

### Ruminal and Fecal Microbial Composition

Analysis of rumen and fecal samples was carried out at Lianchuan Biotechnology Co., Ltd. using 16S rDNA sequencing. DNA from different samples was extracted using the E.Z.N.A. ®Stool DNA kit (D4015, Omega, Inc., USA) according to the manufacturer's instructions. Nuclear-free water was used as a blank. The total DNA was eluted in 50 μl of elution buffer and stored at −80°C until polymerase chain reaction (PCR) analysis was performed at LC-Bio Technology Co., Ltd. (Zhejiang, China).

The 5' ends of the primers were tagged with specific barcodes and universal sequencing primers. PCR amplification was performed in a 25 μl reaction mixture containing 25 ng of template DNA, 12.5 μl of PCR Premix, 2.5 μl of each primer, and PCR-grade water to adjust the final volume. The PCR conditions to amplify prokaryotic 16S fragments were as follows: initial denaturation at 98°C for 30 s; 32 cycles of denaturation at 98°C for 10 s, annealing at 54°C for 30 s, extension at 72°C for 45 s; and a final extension at 72°C for 10 s. The PCR products were confirmed with 2% agarose gel electrophoresis. Throughout the DNA extraction process, ultrapure water was used instead of a sample solution as a negative control to exclude the possibility of false-positive PCR results. The PCR products were purified by AMPure XT beads (Beckman Coulter Genomics, Danvers, MA, USA) and quantified by Qubit (Invitrogen, USA). The amplicon pools were prepared for sequencing and the size and quantity of the amplicon libraries were assessed on an Agilent 2100 Bioanalyzer (Agilent Technologies, USA) and with the KAPA Library Quantification Kit for Illumina sequencing platforms (Kapa Biosciences, Woburn, MA, USA), respectively. The libraries were sequenced on a NovaSeq PE250 platform.

### Statistical Analysis

Data on growth parameters were analyzed using one-way analysis of variance (ANOVA) followed by Duncan's multiple range tests. Alpha diversity index data are presented as the means ± SD and were analyzed by ANOVA with Fisher's least significant difference (LSD) test. Statistical analyses were performed using SPSS version 25.0 for Windows (SPSS Inc., Chicago, Illinois, USA). *P*-value of <0.05 was considered statistically significant.

Samples were sequenced on an Illumina NovaSeq platform according to the manufacturer's recommendations (LC-Bio Technologies). Paired-end reads were assigned to samples based on their unique barcode and truncated by cutting off the barcode and primer sequence. Paired-end reads were merged using FLASH. Quality filtering of the raw reads was performed under specific filtering conditions to obtain high-quality clean tags according to fqtrim (v0.94). Chimeric sequences were filtered using VSEARCH software (v2.3.4). The feature table and feature sequence were obtained after dereplication using DATA2. The alpha and beta diversities were calculated by normalization to the same sequencing depth by randomly removing aligned reads. Then according to the SILVA (release 132) classifier, feature abundance was normalized using the relative abundance of each sample. Alpha diversity was used to analyze the complexity of species diversity for a sample through five indices, including Chao1, observed species, Goods coverage, Shannon and Simpson. All the indices were calculated with QIIME2. Beta diversity was calculated by QIIME2, and the graphs were drawn using the R package. Blast was used for sequence alignment, and the feature sequences were annotated with the SILVA database for each for representative sequences. Other diagrams were generated using the R package (v3.5.2).

## Results

### Growth Performance

The initial weight, final weight, ADG and feed conservation ratio were not significantly different among the diet groups (Table 2). The tendency for total feed intake was higher in the HCB group than in the other groups (0.05 < *P*<*0.10*; Table 1).

**Table 2:** Effects of different doses of *C. butyricum* on growth of fattening goats.

	**Diet^a^**				
**Item**	**CON**	**LCB**	**HCB**	**SEM^b^**	***P-value***
Initial weight, kg	22.28	22.44	23.01	0.38	0.731
Final weight, kg	26.74	25.46	26.79	0.38	0.275
Average daily gain, g	79.70	54.04	67.41	5.70	0.188
Total feed intake, kg	57.11	53.03	62.17	1.58	0.054
Feed conversion ratio, kg/kg	12.80	17.55	16.45	3.73	0.241

a *Diets: CON, control, a basal diet; LCB, low C. butyricum, a basal diet plus 2.0 × 10^8^ CFU/kg; HCB, high C. butyricum, a basal diet plus 1.0 × 10^9^ CFU/kg*.

b *SEM, total standard error of means (n = 8)*.

### Apparent Digestibility of Nutrients

The results of the apparent digestibility of different diet groups are shown in Table 3. Different doses of *C. butyricum* had no significant effect on the apparent digestibility of DM, CP, EE, NDF, ADF, fecal N and urinary N in fattening goats (*P* > *0.05*). No significant difference in fecal N and urine N was observed among the treatment groups (Table 3).

**Table 3:** Effects of different doses of *C. butyricum* on apparent digestibility of fattening goats.

	**Diet^a^**				
**Item^b^**	**CON**	**LCB**	**HCB**	**SEM^c^**	***P-value***
**Apparent digestibility %**
DM	78.02	83.68	80.52	2.01	0.536
CP	86.62	89.98	89.09	1.24	0.537
EE	87.42	91.34	87.97	1.40	0.488
NDF	72.04	79.38	73.93	2.72	0.540
ADF	77.71	77.75	72.74	2.45	0.652
**N metabolism, g/d**
NI	22.78	21.99	23.70	0.69	0.625
Fecal N	3.15	2.14	2.61	0.30	0.413
Urine N	3.07	2.21	3.14	0.25	0.246
NAB	19.64	19.86	21.09	0.66	0.641
NR	16.56	17.65	17.96	0.63	0.656
NPU	73.26	79.72	75.95	1.65	0.286

a *Diets: CON, control, a basal diet; LCB, low C. butyricum, a basal diet plus 2.0 × 10^8^ CFU/kg; HCB, high C. butyricum, a basal diet plus 1.0 × 10^9^ CFU/kg*.

b *DM, dry matter, CP, crude protein; EE, ether extract; NDF, neutral detergent fiber; ADF, acid detergent fiber; NI, nitrogen intake; NAB, nitrogen absorption; NR, nitrogen retention; NPU, net protein utilization*.

c *SEM, total standard error of means (n = 8)*.

### Plasma Metabolites

*C. butyricum* supplementation had no significant effect on the concentrations of serum BUN, CREA, GLU, ALT, AST, TP, ALB, GLB, TG, T-CHOL, HDL and LDL (Table 4).

**Table 4:** Effects of different doses of *C. butyricum* on serum metabolites of fattening goats.

	**Diet^a^**				
**Item^b^**	**CON**	**LCB**	**HCB**	**SEM^c^**	***P-value***
BUN, mmol/L	7.99	8.65	9.29	0.39	0.411
CREA, mmol/L	43.44	51.05	47.31	2.39	0.449
GLU, mmol/L	3.31	3.33	3.69	0.22	0.738
ALT, mmol/L	35.38	31.75	34.63	1.59	0.638
AST, mmol/L	124.25	124.00	135.25	3.74	0.392
TP, mmol/L	64.33	67.06	69.53	2.24	0.658
ALB, mmol/L	34.53	35.70	36.48	0.87	0.675
GLB, mmol/L	29.80	31.36	33.05	1.49	0.693
TG, mmol/L	0.34	0.34	0.32	0.02	0.925
T-CHOL, mmol/L	1.86	2.15	2.41	0.14	0.301
HDL, mmol/L	0.94	1.03	1.10	0.05	0.526
LDL, mmol/L	0.79	0.91	1.04	0.08	0.447

a *Diets: CON, control, a basal diet; LCB, low C. butyricum, a basal diet plus 2.0 × 10^8^ CFU/kg; HCB, high C. butyricum, a basal diet plus 1.0 × 10^9^ CFU/kg*.

b *BUN, blood urea nitrogen; CREA, creatinine; GLU, glucose; ALT, alanine transaminase; AST, aspartate trans-aminase; TP, total protein; ALB, albumin; GLB, globulin; TG, triglyceride; CHOL, cholesterol; HDL, high-density lipoprotein; LDL, low-density lipoprotein*.

c *SEM; total standard error of means (n = 8)*.

### Ruminal Fermentation

As shown in Table 5, *C. butyricum* supplementation affected rumen fermentation including ruminal pH (*P* = *0.003*). Ruminal pH was lower in the LCB group, but higher in the CON and HCB groups (*P*<*0.01*). The contents of total VFA (*P* = *0.057*) and propionate (*P* = *0.089*) tended to decrease in the HCB group compared with the CON group. No significant difference was observed in the concentrations of NH_3_-N, acetate and butyrate.

**Table 5:** Effects of different doses of *C. butyricum* on rumen fermentation of fattening goats.

	**Diet^c^**				
**Item**	**CON**	**LCB**	**HCB**	**SEM^d^**	***P-value***
Ruminal pH	5.75^a^	5.37^b^	5.92^a^	0.08	<0.003
NH_3_-N^3^, mg/dl	26.79	30.36	29.94	1.90	0.742
**Ruminal VFA** ^**4**^ **, mmol/L**
Total VFA	77.10	76.53	61.54	3.16	0.057
Acetate	47.31	52.02	42.71	2.12	0.212
Propionate	20.93	17.95	13.03	1.52	0.089
Butyrate	8.86	5.51	5.74	1.11	0.426

ab * with row, different superscripts indicate differences between treatments (P ≤ 0.05)*.

c *Diets: CON, control, a basal diet; LCB, low C. butyricum, a basal diet plus 2.0 × 10^8^ CFU/kg; HCB, high C. butyricum, a basal diet plus 1.0 × 10^9^ CFU/kg*.

d *SEM, total standard error of means (n = 4)*.

### Ruminal and Fecal Microbial Composition

**Figure 1:**
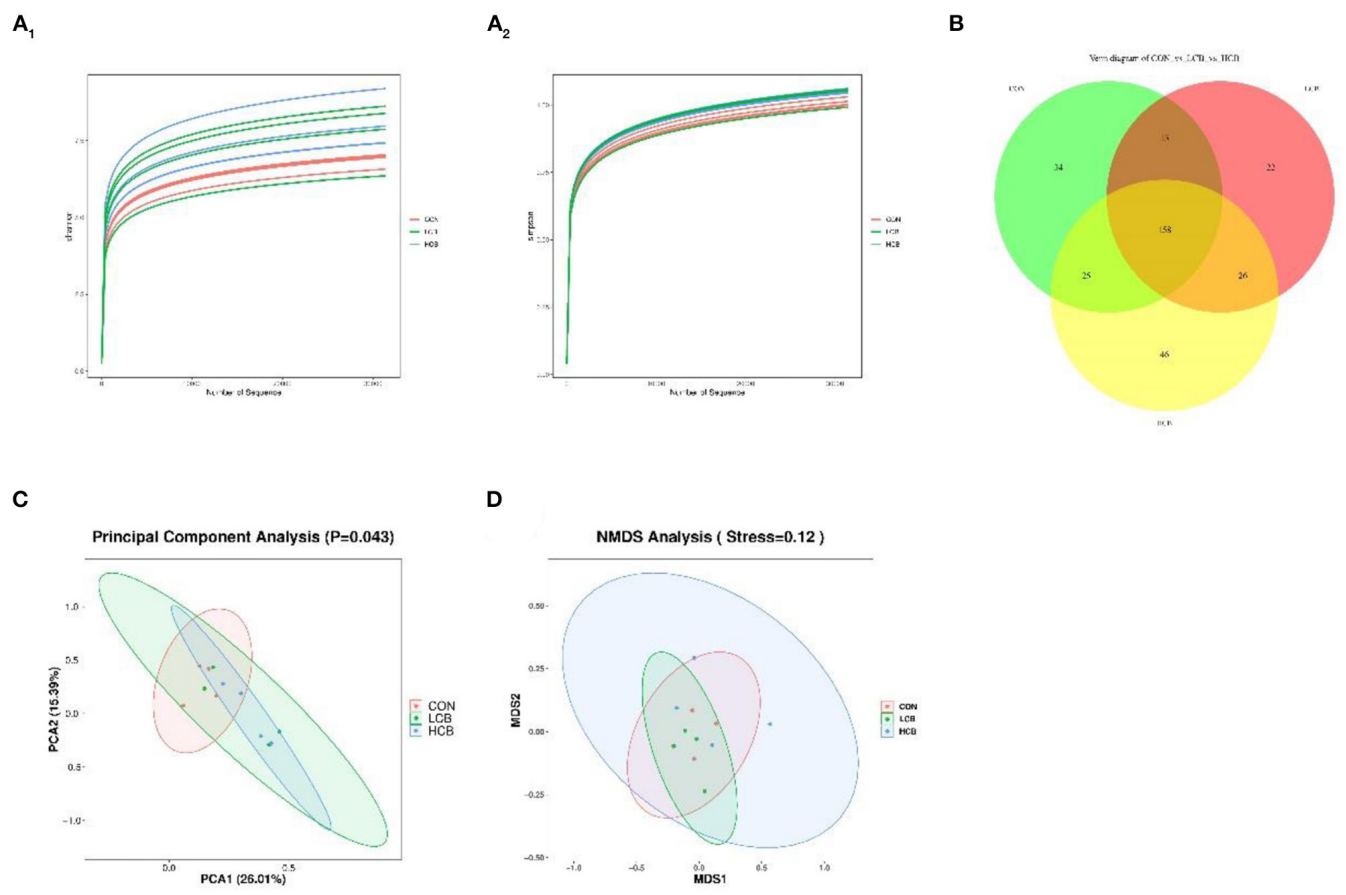
The α- and β-diversity of rumen bacterial communities in fattening goats fed *C. butyricum*. Shannon **(A**_**1**_**)** and Simpson **(A**_**2**_**)** curves of rumen microbiome of fattening goats are shown. Unique and shared rumen feature among CON, LCB, and HCB groups **(B)** shown on Venn diagrams. Rumen microbial structure among the three groups was estimated by the Principal Component Analysis **(C)** and Nonmetric Multidimensional Scaling Analysis **(D)**.

**Figure 2:**
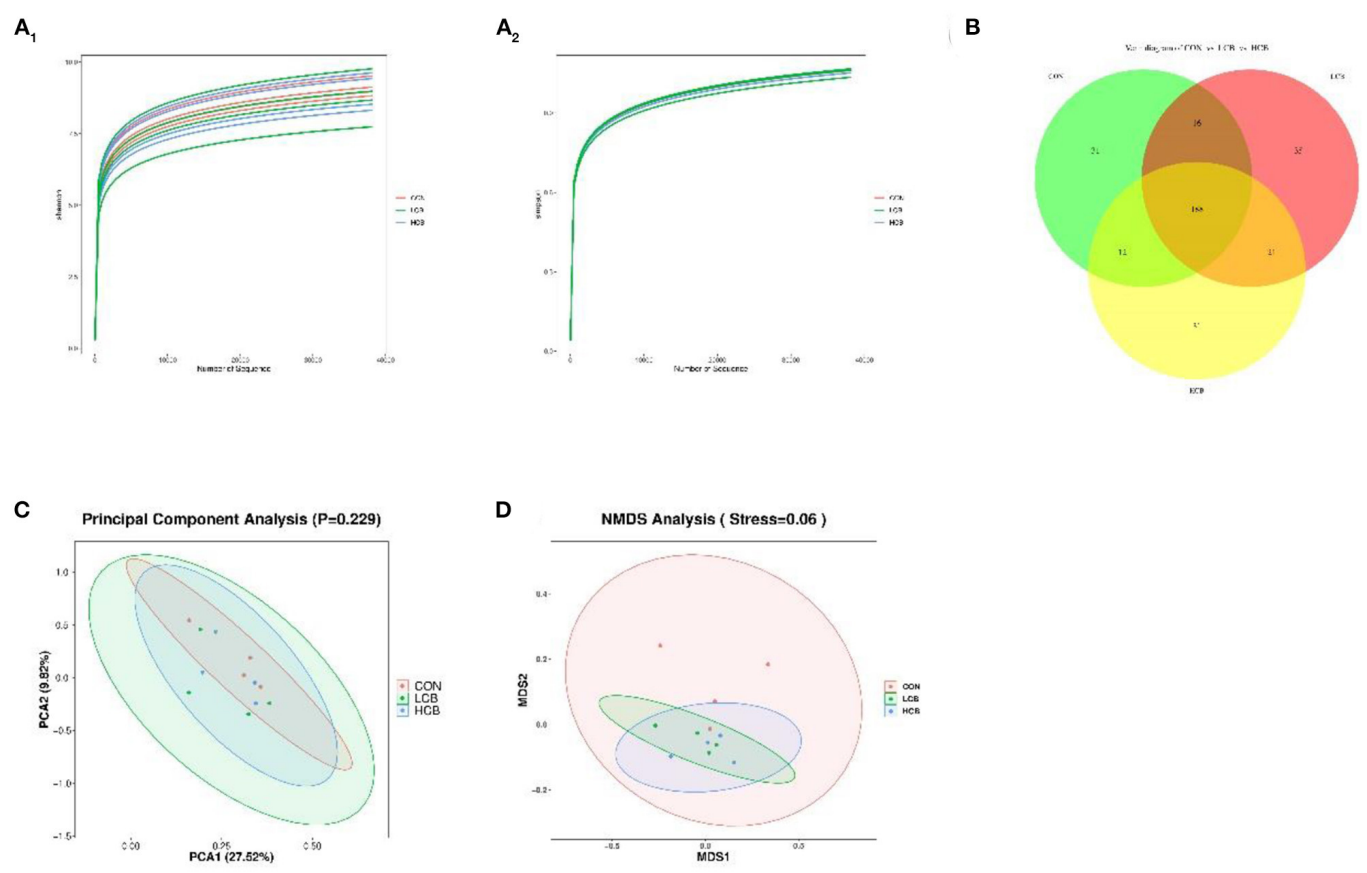
The α- and β-diversity of fecal bacterial communities in fattening goats fed *C. butyricum*. Shannon **(A**_**1**_**)** and Simpson **(A**_**2**_**)** curves of fecal microbiome of fattening goats are shown. Unique and shared rumen feature among CON, LCB, and HCB groups **(B)** shown on Venn diagrams. Fecal microbial structure among the three groups was estimated by the Principal Component Analysis **(C)** and Nonmetric Multidimensional Scaling Analysis **(D)**.

To further understand the effects of dietary supplementation with *C. butyricum* on the rumen and fecal microbiota, 16S rDNA sequencing was performed. A total of 548,229 and 823,673 clean tags were obtained from rumen fluid and fecal samples, respectively. The CON, LCB and HCB groups of all samples based on Shannon and Simpson dilution curve analysis were observed in all the samples (Figures 1A_1_,A_2_, 2A_1_,A_2_), suggesting that the sequencing depth was sufficient to accurately characterize the rumen and fecal bacterial composition. A total of 362 sequences were found at the genus level of rumen microorganisms, of which 158 were common. A total of 334 sequences were found at the genus level of fecal microorganisms, including 188 common sequences, indicating the existence of a large common microbiome (Figures 1B, 2B). Principal component analysis (PCA) of rumen microflora revealed significant differences in the microbial community richness and diversity between and within groups (*P* = 0.043; *P* < 0.05), but PCA of the fecal microflora showed no significant differences (*P* = 0.229; *P* > 0.05) (Figures 1C, 2C). Another bacterial community composition of the rumen and fecal microflora is presented in Figures 1D, 2D. Nonmetric multidimensional scaling (NMDS) analysis showed that rumen microbial communities were partially representative among the three groups (0.1 < *P* = 0.12 <0.20). However, the composition of fecal microflora was highly representative (0.05 < *P* = 0.06 <0.10).

**Figure 3:**
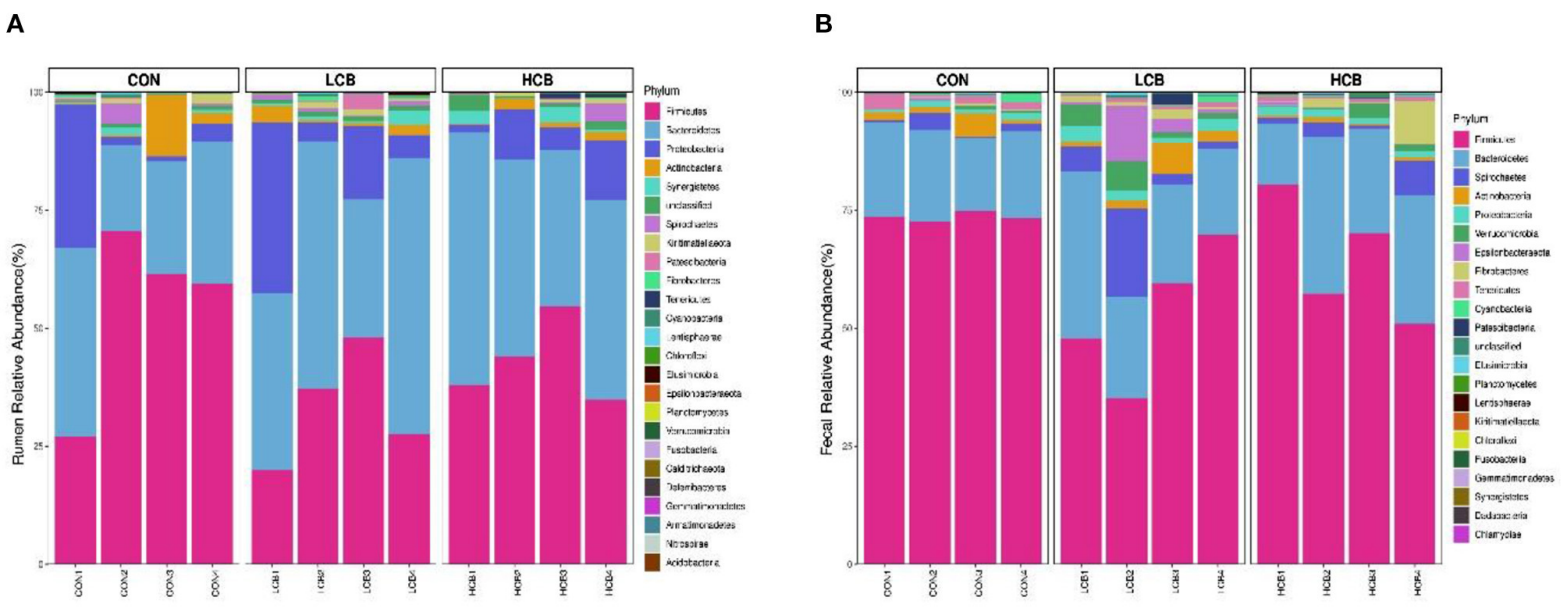
Stacked bar graph displays a comparison of microbial relative abundance (%) at phylum level among main effects of treatments in rumen **(A)** and fecal **(B)** of fattening goats. The label CON denotes without prebiotics, the label LCB denotes low-dose *C. butyricum*, and the label HCB denotes high-dose *C. butyricum* for each sample. The letters (1,2,3 and 4) denote replicate samples. CON: 0 CFU *C. butyricum* per kilogram feed, LCB: 2.0 × 10^8^ CFU *C. butyricum* per kilogram feed, HCB: 1.0 × 10^9^ CFU *C. butyricum* per kilogram feed.

**Figure 4:**
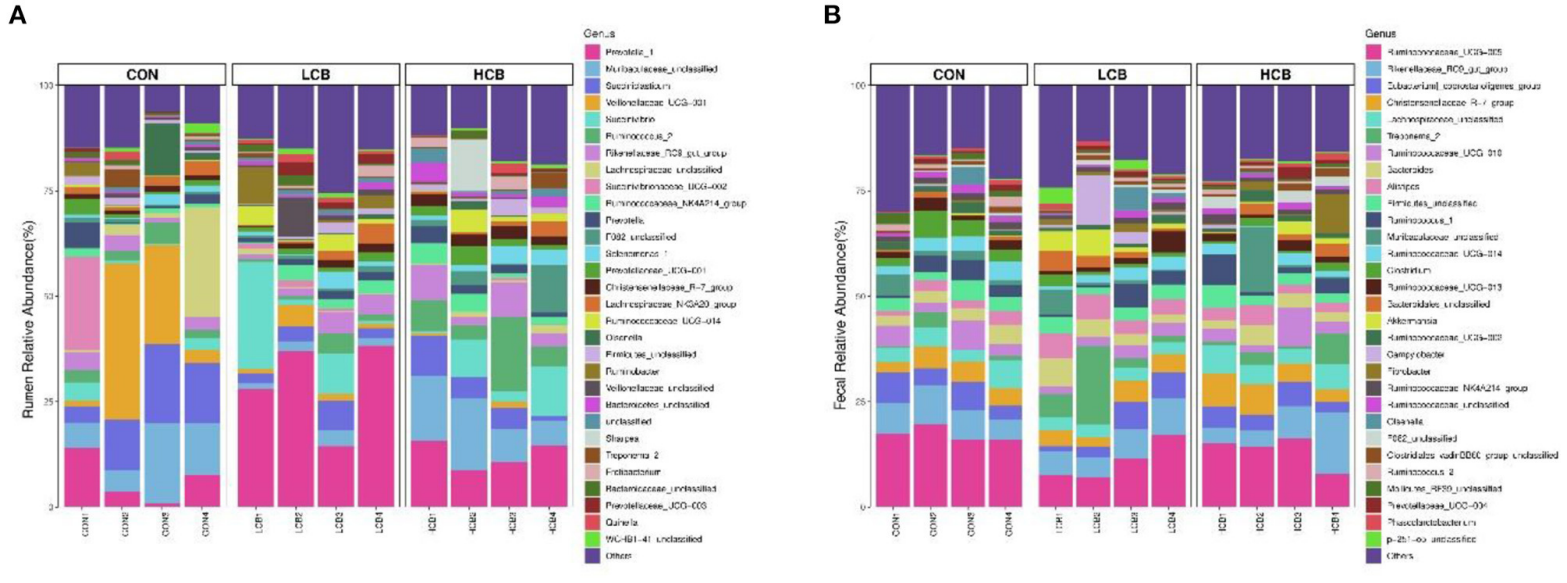
Stacked bar graph displays a comparison of microbial relative abundance (%) at genus level among main effects of treatments in rumen **(A)** and fecal **(B)** of fattening goats. The label CON denotes without prebiotics, the label LCB denotes low-dose *C. butyricum*, and the label HCB denotes high-dose *C. butyricum* for each sample. The letters (1,2,3 and 4) denote replicate samples. CON: 0 CFU *C. butyricum* per kilogram feed, LCB: 2.0 × 10^8^ CFU *C. butyricum* per kilogram feed, HCB: 1.0 × 10^9^ CFU *C. butyricum* per kilogram feed.

**Figure 5:**
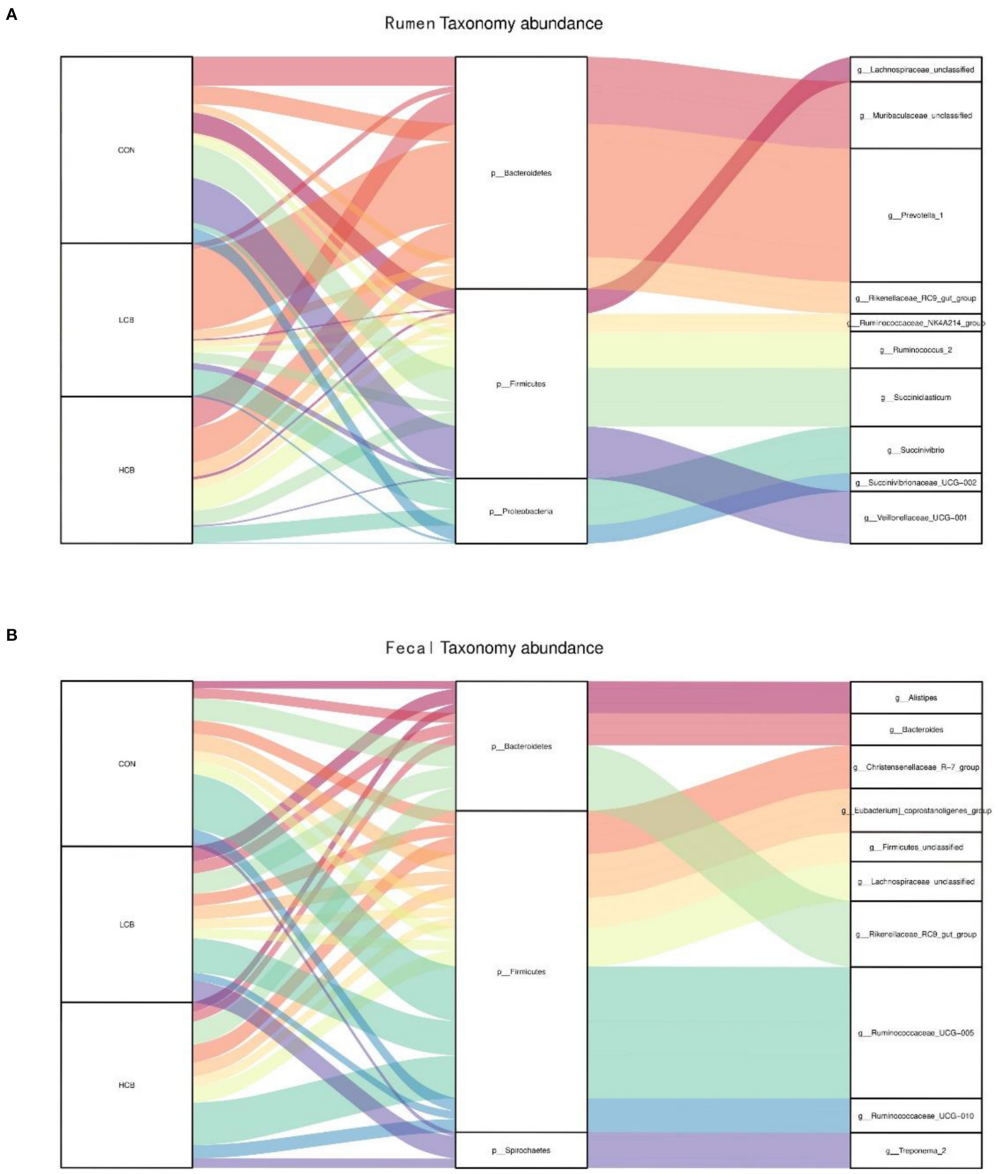
Sankey plot displays a comparison of microbial relative abundance (%) at genus level among main effects of treatments in rumen **(A)** and fecal **(B)** of fattening goats. The label CON denotes without prebiotics, the label LCB denotes low-dose *C. butyricum*, and the label HCB denotes high-dose *C. butyricum* for each sample. CON: 0 CFU *C. butyricum* per kilogram feed, LCB: 2.0 × 10^8^ CFU *C. butyricum* per kilogram feed, HCB: 1.0 × 10^9^ CFU *C. butyricum* per kilogram feed.

A total of 25 phyla were identified in the rumen fluid samples. There were seven phyla with an abundance of ≥ 1.0% in each group, including *Firmicutes, Bacteroidetes, Proteobacteria Actinobacteria, Synergistetes, Unclassified* and *Spirochaetes* (*P* > 0.05) (Figures 3A, **5A**, Table 6). At the genus level, the major microorganisms were *Prevotella1, Muribaculaceae unclassified, Succiniclasticum* and *Veillonellaceae UCG-001*. A notable change in the rumen microbiota composition was observed in response to *C. butyricum* following treatment with different doses of groups. Generally, with significantly different abundances between treatments, *C. butyricum* supplementation significantly increased the relative abundance of *Prevotella1, Muribaculaceae unclassified, Christensenellaceae R-7group* and *Prevotellaceae UCG-003* to varying degrees (*P* < 0.05). Interestingly, the relative abundance of *Succiniclasticum* was significantly lower in the LCB and HCB groups than in the CON group (*P* = *0.047*), and the LCB group had the lowest relative abundance (Figures 4A, 5A, Table 7).

**Table 6:** Effects of different doses of *C. butyricum* on phylum relative rumen microbiota abundances (>1.0%) of fattening goats.

	**Diet^a^**				
**Item**	**CON**	**LCB**	**HCB**	**SEM^b^**	***P-value***
*Firmicutes*	54.65	33.19	42.8	4.51	0.150
*Bacteroidetes*	28.05	44.42	42.66	3.54	0.109
*Proteobacteria*	9.22	15.1	7.42	3.34	0.661
*Actinobacteria*	3.94	1.72	1.3	1.02	0.569
*Synergistetes*	0.64	1.18	1.63	0.33	0.518
*unclassified*	0.57	0.99	1.59	0.25	0.273
*Spirochaetes*	1.29	0.70	1.09	0.42	0.867

a *Diets: CON, control, a basal diet; LCB, low C. butyricum, a basal diet plus 2.0 × 10^8^ CFU/kg; HCB, high C. butyricum, a basal diet plus 1.0 × 10^9^ CFU/kg*.

b *SEM, total standard error of means (n = 4)*.

**Table 7:** Effects of different doses of *C. butyricum* on genus relative rumen microbiota abundances (>1.0%) of fattening goats.

	**Diet^c^**				
**Item**	**CON**	**LCB**	**HCB**	**SEM^d^**	***P-value***
*Prevotella_1*	6.61^b^	29.47^a^	12.44^b^	3.51	<0.005
*Muribaculaceae_unclassified*	10.57^a^	2.26^b^	11.52^a^	1.80	<0.050
*Succiniclasticum*	12.24^a^	3.80^b^	5.21^b^	1.59	<0.047
*Veillonellaceae_UCG-001*	16.24	2.24	0.58	3.35	0.101
*Succinivibrio*	1.99	9.21	5.80	2.13	0.422
*Ruminococcus_2*	3.05	1.95	8.36	1.32	0.096
*Rikenellaceae_RC9_gut_group*	3.05	3.22	5.35	0.70	0.362
*Lachnospiraceae_unclassified*	7.48	0.54	1.02	2.09	0.351
*Succinivibrionaceae_UCG-002*	5.59	0.83	0.21	1.83	0.463
*Ruminococcaceae_NK4A214_group*	1.12	2.35	2.97	0.43	0.215
*Prevotella*	2.22	1.62	2.24	0.46	0.847
*F082_unclassified*	0.73	0.80	4.11	0.89	0.219
*Selenomonas_1*	1.27	1.61	2.62	0.42	0.433
*Prevotellaceae_UCG-001*	1.60	1.36	2.35	0.36	0.537
*Christensenellaceae_R-7_group*	1.18^b^	1.41^b^	2.70^a^	0.25	<0.008
*Lachnospiraceae_NK3A20_group*	1.98	1.86	1.36	0.42	0.844
*Ruminococcaceae_UCG-014*	0.31	2.49	2.17	0.55	0.230
*Olsenella*	3.58	0.40	0.60	1.02	0.396
*Firmicutes_unclassified*	1.29	1.23	1.85	0.30	0.681
*Ruminobacter*	0.82	3.08	0.13	0.76	0.269
*Veillonellaceae_unclassified*	0.61	3.04	0.25	0.74	0.266
*Bacteroidetes_unclassified*	0.53	0.94	2.25	0.35	0.101
*unclassified*	0.57	0.99	1.59	0.25	0.273
*Sharpea*	0.00	0.01	3.09	1.01	0.390
*Treponema_2*	1.26	0.47	1.07	0.42	0.759
*Fretibacterium*	0.47	0.94	1.36	0.32	0.561
*Bacteroidaceae_unclassified*	0.79	1.25	0.60	0.22	0.522
*Prevotellaceae_UCG-003*	0.33^b^	1.70^a^	0.37^b^	0.27	<0.038
*Quinella*	0.53	1.11	0.65	0.25	0.654
*Clostridium^e^*	0.14	0.22	0.05	0.07	0.612

ab * with row, different superscripts indicate differences between treatments (P ≤ 0.05)*.

c *Diets: CON, control, a basal diet; LCB, low C. butyricum, a basal diet plus 2.0 × 10^8^ CFU/kg; HCB, high C. butyricum, a basal diet plus 1.0 × 10^9^ CFU/kg*.

d *SEM, total standard error of means (n = 4)*.

e *Special observation on relative abundance (<1.0%) of supplemental C. butyricum in rumen*.

*Firmicutes, Bacteroidetes* and *Spirochaetes* were the three major phyla (Figures 3B, 5B, Table 8). At the genus level, *Ruminococcaceae UCG005, Christensenellaceae R7group, Ruminococcaceae UCG010* and *Bacteroides* were the dominant species. *Clostridium* and *F082 unclassified* decreased significantly (*P* < 0.05) after treatment with *C. butyricum* and did not become the dominant bacteria. *Ruminococcaceae UCG005* showed a significant downward trend (*P* = *0.081*), but *Alistipes* (*P* = *0.062*) and *Akkermansia* (*P* = *0.069*) showed the opposite trend (Figures 4B, 5B, Table 9).

**Table 8:** Effects of different doses of *C. butyricum* on phylum relative fecal microbiota abundances (>1.0%) of fattening goats.

	**Diet^a^**				
**Item**	**CON**	**LCB**	**HCB**	**SEM^b^**	***P-value***
*Firmicutes*	73.58	53.12	64.71	3.92	0.090
*Bacteroidetes*	18.32	23.97	23.87	1.94	0.436
*Spirochaetes*	1.48	6.99	3.09	1.48	0.324
*Actinobacteria*	2.15	2.81	0.65	0.55	0.278
*Proteobacteria*	1.13	2.25	1.53	0.20	0.058
*Verrucomicrobia*	0.29	3.29	1.29	0.58	0.082
*Epsilonbacteraeota*	0.16	4.00	0.22	0.96	0.176
*Tenericutes*	1.76	0.77	0.78	0.73	0.127
*Fibrobacteres*	0.23	1.19	2.87	0.15	0.366

a *Diets: CON, control, a basal diet; LCB, low C. butyricum, a basal diet plus 2.0 × 10^8^ CFU/kg; HCB, high C. butyricum, a basal diet plus 1.0 × 10^9^ CFU/kg*.

b *SEM, total standard error of means (n =4)*.

**Table 9:** Effects of different doses of *C. butyricum* on genus relative fecal microbiota abundance (>1.0%) of fattening goats.

	**Diet^c^**				
**Item**	**CON**	**LCB**	**HCB**	**SEM^d^**	***P-value***
*RuminococcaceaeUCG005*	17.34	10.84	13.45	1.23	0.081
*RikenellaceaeRC9gutgroup*	6.98	6.45	7.40	0.87	0.922
*ChristensenellaceaeR7group*	4.21	3.82	5.61	0.48	0.306
*Eubacteriumcoprostanoligenesgroup*	5.32	4.18	4.29	0.57	0.704
*Lachnospiraceaeunclassified*	4.26	3.02	5.15	0.43	0.122
*RuminococcaceaeUCG010*	4.17	2.47	4.15	0.65	0.521
*Alistipes*	2.32	4.69	3.02	0.44	0.062
*Firmicutesunclassified*	3.21	2.99	3.21	0.32	0.958
*Treponema2*	1.36	6.98	2.94	1.49	0.312
*Bacteroides*	3.09	3.91	3.12	0.41	0.701
*Ruminococcus1*	3.04	2.53	3.22	0.64	0.918
*RuminococcaceaeUCG014*	3.22	2.09	1.75	0.34	0.186
*Muribaculaceaeunclassified*	2.35	1.96	4.07	1.25	0.799
*Clostridium*	3.79^a^	1.37^b^	1.29^b^	0.48	<0.036
*RuminococcaceaeUCG013*	1.92	2.07	1.56	0.36	0.861
*Akkermansia*	0.15	3.28	1.29	0.58	0.069
*RuminococcaceaeNK4A214group*	1.78	1.21	1.60	0.12	0.782
*RuminococcaceaeUCG002*	1.12	1.77	0.16	0.19	0.493
*Olsenella*	0.74	2.41	1.83	0.53	0.509
*Bacteroidalesunclassified*	1.42	0.92	1.09	0.40	0.236
*Ruminococcaceaeunclassified*	0.23	1.19	2.87	0.16	0.486
*Fibrobacter*	0.16	3.99	0.21	0.73	0.366
*Campylobacter*	1.31	0.55	0.65	0.96	0.177
*Ruminococcus2*	0.37	0.60	1.98	0.17	0.150
*F082unclassified*	0.86^b^	0.77^b^	1.24^a^	0.29	<0.034
*ClostridialesvadinBB60groupunclassified*	1.46	0.47	0.58	0.14	0.350
*MollicutesRF39unclassified*	0.72	0.66	1.12	0.21	0.104

ab * with row, different superscripts indicate differences between treatments (P ≤ 0.05)*.

c *Diets: CON, control, a basal diet; LCB, low C. butyricum, a basal diet plus 2.0 × 10^8^ CFU/kg; HCB, high C. butyricum, a basal diet plus 1.0 × 10^9^ CFU/kg*.

d *SEM, total standard error of means (n = 4)*.

## Discussion

To the best of our knowledge, this is the first study to examine the effects of dietary *C. butyricum* supplementation in a high-concentrate diet on growth performance in fattening goats. The results showed no significant improvement in daily gain, which is consistent with the results for weaned piglets, in which no significant improvement in growth performance was found during the 21-day observation period (27, 28). However, we found that most of the studies on the growth performance of *C. butyricum* in monogastric animals reported beneficial results (29). Dietary *C. butyricum* was able to increase intestinal villus height and crypt depth in monogastric animals, indicating that intestinal cell absorption capacity was improved. Probiotic fermentation in the cecum, provided the host with large amounts of VFAs, amino acids, vitamins, and other metabolites (30, 31). These rich nutrients pass through vector genes such as solute vector family 7 member 11(SLC7A11), low-density lipoprotein-associated protein 2(LRP2), transporter 2(TSPO2), hemoglobin subunit μ(HBM), upregulation of hemoglobin subunit α1(HBA1) and ENSGALG00000050921(ultralong-chain fatty acid CoA ligase activity) to increase average daily gain and improve growth performance. Meanwhile, *C. butyricum* secreted more butyric acid and propionic acid in the rumen to provide energy for the body, and the effects of the growth performance of ruminants were also positive (32, 33). Therefore, based on the positive effects of numerous *C. butyricum* in animal production, our interest in its application and exploration has increased. However, in contrast to the above studies, the results of growth performance in this experiment were not consistent with the above studies and found that the content of VFA decreased during rumen fermentation. This may affect the transport of bacteria-produced nutrients to the gut when *C. butyricum* and its metabolites are fermented with high carbohydrate levels in the rumen.

The digestibility of nutrients in feed is crucial for the growth and development of ruminants. It reflects the digestion and absorption of nutrients by the animal's body. Previous studies have shown that the addition of probiotics to diets improves the digestibility of DM, CP, EE and fiber (32, 33). Dietary supplementation with *C. butyricum* promotes the activities of intestinal digestive enzymes, including trypsin and α -amylase, resulting in increased starch and CP digestibility (4). Meanwhile, *C. butyricum* can increase the concentrations of organic acids such as butyric acid and acetic acid in the body, reduce the pH in the intestinal tract, and improve the digestion of nutrients (34). In a study of heat-stressed goats, *C. butyricum* was reported to be effective in increasing the digestibility of DM, NDF and ADF (32). This may be related to rumen flora activity. Studies have shown that the pH in the rumen is between 6.0 and 7.0, which is the ideal state fiber-digesting and acid-sensitive bacteria, and the activity of *C. butyricum* decreases in a lower pH environment (35). According to our results, the pH values were all <6.0, which may be the result of high carbohydrate fermentation in the rumen, thus affecting the rumen metabolism and nutrient digestibility.

Interestingly, the rumen environment of the *C. butyricum* group was improved. The rumen pH was significantly increased with the increase in *C. butyricum* dose in the HCB group compared with the LCB group. Recent studies have shown that *C. butyricum* protects intestinal barrier function and the immune response by regulating the microbiome (12, 27, 36, 37). To our knowledge, this is the first study to investigate the microbiota of goats fed *C. butyricum*. It was found that the 8-week high-concentrate diet did not cause acidosis or subacute acidosis in the test animals. This implies that the optimization of the rumen pH in the treatment group can also be attributed to the influence on the production and transport of VFA. Our results showed that *C. butyricum* reduced total volatile acid production, which was mainly influenced by changes in the rumen microbiota. *Succiniclasticum* (from Firmicutes), as the dominant rumen succinic acid-producing bacteria, plays a crucial role in the conversion of succinic acid to propionic acid (38). It was reported that a decrease in propionic acid production was accompanied by a decrease in the relative abundance of *Succiniclasticum* (39), which is consistent with the results obtained in the present study. Furthermore, the propionic acid can be converted to GLU in the liver through rumen wall gluconeogenesis. The increase in blood GLU plays an important role in the fattening period of goats. However, the results of the current trial showed that there were no significant changes in blood GLU content among all treatment groups, which may be related to the change of in the relative abundance of *Succiniclasticum* in the rumen, and the concentration of VFA in the rumen was not improved without a significant influence on the growth of goats.

It was found that *C. butyricum* alters the abundance of *Prevotella, Muribaculaceae* and *Succiniclasticum*. Similar results have been reported in monogastric animals: dietary supplementation with *C. butyricum* increases the relative abundance of *Prevotella, Ruminococcaceae, Megasphaera*, and other species, consumes lactic acid and converts it to VFA (acetic acid, propionic acid, butyric acid, etc.) (27). An appropriate amount of *C. butyricum* can also promote the colonization of probiotics in the digestive tract, consolidate the intestinal barrier protection mechanism, and fight against the invasion of harmful bacteria (12). The positive impact of the above studies has given us confidence in the study of the effects of *C. butyricum* on ruminants. In addition, studies have shown that supplementation with 5 g/100 kg body weight of *C. butyricum* and its combination with yeast could improve the rumen VFA concentration (32), while feeding with 1.5 or 3.0 g/100 kg body weight did not affect the concentration of VFA in the rumen of calves (40), meaning that a high dose of *C. butyricum* was beneficial for improving the rumen fermentation function. Combined with the results of the current study, one possibility for the relative abundance of *C. butyricum* being low in the rumen was that dietary supplementation with *C. butyricum* formed an antagonistic relationship with the dominant flora. Another may be that the rumen environment is not the optimal environment for *C. butyricum*.

*Fibrolytic bacteria* are one of the most important bacteria in the rumen. They regulate the production and distribution of VFAs and affect the digestibility of host fiber by secreting cellulase. Previous studies have found that several phyla, such as *Proteus, Tenikoot* and TM7, and several bacterial genera, including *Anaerobe, Campylobacter* and *Clostridium*, are associated with the apparent digestibility of crude fiber in pigs (41). However, the change in rumen microbiota did not affect the performance in the present study. Previous reports found that rumen bacteria were positively correlated with the gene expression level and VFA content in rumen epithelial cells in adult beef cattle (42). In addition, altered VFAs also play a key role in gene expression in rumen epithelial cells (43). These observations suggest that changes in rumen microflora can regulate rumen homeostasis. However, the causal relationship and physiological mechanisms affecting rumen ecological balance remain unclear.

The fecal *Clostridium* content was significantly higher in the CON group than in the treatment groups. This result is inconsistent with the findings from other studies, in which *C. butyricum* addition to the diet of piglets did not change the fecal *Clostridium* or *C. butyricum* content (28). *C. butyricum* was also found to significantly increase acetic acid-producing bacteria in the intestinal tract, as shown by the increased production of acetic acid by *Prevotella, Selenomonas* and *Megasphaera* through the *methylmalonyl-CoA* and butyryl-CoA: *acetate COA*-transferase pathways, converting it directly or indirectly. To meet the butyric acid requirements of intestinal cells, it is speculated that the *C. butyricum* microbiota could undergo internal transformation (28). This may be related to the metabolic activities of *C. butyricum* in the rumen. According to our results, the relative abundance of *Prevobacteria* in the rumen supplemented with *C. butyricum* was significantly increased, which was consistent with the above results. However, *Clostridium* was not the dominant flora in the rumen (relative abundance <1%). In addition, the number of *Alispeties* (from Proteobacteria) tended to increase in the feces of the supplemented group. Related studies have shown that *Alispeties* bacteria are involved in the expression of enzymes associated with propionic acid synthesis (44), and induce low-grade inflammation caused by enterotoxins secreted by intestinal bacteria (45). These processes may be associated with harmful bacteria competing for nutrients and affecting the proportion of *Clostridium* in the intestinal tract. Therefore, further studies are needed to fully understand the activities of *C. butyricum* in the digestive tract of ruminants as well as the metabolite levels of *C. butyricum* in the rumen and intestinal tract and colonization in different parts of the digestive system.

In summary, the current study indicated that supplementation with a high dose of *C. butyricum* in high-concentrate diets can affect the rumen fermentation process by regulating the abundance of rumen microflora without affecting growth performance. Therefore, our future studies should combine molecular biology and omics analyses to reveal the interaction mechanism between *C. butyricum* and goat performance.

## Conclusion

Dietary supplementation with high dose *C. butyricum* in high-concentrate diets can change the concentration of VFA by regulating the abundance of rumen bacterial communities to affect rumen fermentation and help maintain rumen homeostasis. However, there were no negative effects on growth performance, apparent digestibility or serum metabolites in fattening goats. Overall, the effect of *C. butyricum* as a potential probiotic feed additive on the growth performance of fattening goats remains to be further explored.

## Data Availability Statement

The data presented in the study are deposited in the NCBI Sequence Read Archive (SRA), accession number PRJNA815468 and PRJNA815470.

## Ethics Statement

The animal study was reviewed and approved by Institutional Animal Care and Use Committee of Northeast Agricultural University (Harbin, China) (Protocol number: NEAU- [2011]-9). Written informed consent was obtained from the owners for the participation of their animals in this study.

## Author Contributions

CZ, YY, and YS conceptualized and designed the study. CZ conducted animal trials, analyzed data, and drafted original manuscript. YY and JW performed laboratory experiments. YS and YZ reviewed and provided critical comments on the manuscript. All authors read and approved the final manuscript.

## Funding

This study was financially supported by Research and Demonstration of Water-saving and Efficient Production and Utilization Technology of High-quality Forage Material (KJXM-EEDS 2020010-04) and China Agriculture Research System of MOF and MARA.

## Conflict of Interest

The authors declare that the research was conducted in the absence of any commercial or financial relationships that could be construed as a potential conflict of interest.

## Publisher's Note

All claims expressed in this article are solely those of the authors and do not necessarily represent those of their affiliated organizations, or those of the publisher, the editors and the reviewers. Any product that may be evaluated in this article, or claim that may be made by its manufacturer, is not guaranteed or endorsed by the publisher.
